# Alzheimer’s disease as a systems network disorder: chronic stress/dyshomeostasis, innate immunity, and genetics

**DOI:** 10.18632/aging.103883

**Published:** 2020-09-21

**Authors:** Alexei Kurakin, Dale E. Bredesen

**Affiliations:** 1 Department of Neurology, David Geffen School of Medicine, University of California Los Angeles, Los Angeles, CA 90095, USA; 2 Department of Molecular and Medical Pharmacology, David Geffen School of Medicine, University of California Los Angeles, Los Angeles, CA 90095, USA; 3 Buck Institute for Research on Aging, Novato, CA 94945, USA

**Keywords:** Alzheimer’s disease, neurodegeneration, complex chronic disorder, network biology, systems biology

## Abstract

Ineffective results of clinical trials of over 200 anti-Alzheimer's drug candidates, with a 99.6% attrition rate, suggest that the current paradigm of Alzheimer's disease (AD) may be incomplete, necessitating exploration of alternative and complementary frameworks.

Using algorithms for hypothesis independent search and expert-assisted synthesis of heterogeneous data, we attempted to reconcile multimodal clinical profiles of early-stage AD patients and accumulated research data within a parsimonious framework. Results of our analysis suggest that Alzheimer’s may not be a brain disease but a progressive system-level network disorder, which is driven by chronic network stress and dyshomeostasis. The latter can be caused by various endogenous and exogenous factors, such as chronic inflammatory conditions, infections, vascular dysfunction, head trauma, environmental toxicity, and immune disorders. Whether originating in the brain or on the periphery, chronic stress, toxicity, and inflammation are communicated to the central nervous system (CNS) via humoral and neural routes, preferentially targeting high-centrality regulatory nodes and circuits of the nervous system, and eventually manifesting as a neurodegenerative CNS disease.

In this report, we outline an alternative perspective on AD as a systems network disorder and discuss biochemical and genetic evidence suggesting the central role of chronic tissue injury/dyshomeostasis, innate immune reactivity, and inflammation in the etiopathobiology of Alzheimer’s disease.

## INTRODUCTION

Alzheimer’s disease has become a global epidemic, rapidly advancing in the last decade to become the 5^th^ leading cause of death globally and the 3^rd^ leading cause of death in high-income countries [1]. There is no cure for AD, and the few currently approved drugs provide only modest and transient symptomatic benefits.

The neuropathological hallmarks of Alzheimer’s disease include extracellular deposits of amyloid-β (Aβ) peptide and intracellular neurofibrillary tangles comprising hyperphosphorylated protein tau [2, 3]. The amyloid cascade hypothesis has been the cornerstone paradigm that guided AD research and drug development efforts for decades, amassing impressive amounts of supporting evidence [4]. The hypothesis postulates that the sequence of events leading to AD begins with the overproduction and accumulation of amyloid-β, followed by neuroinflammation, deposition of neurofibrillary tau tangles, the onset of synaptic and neuronal dysfunction and loss, and eventually overt brain atrophy [4, 5].

Unfortunately, more than 200 drug candidates targeting various key aspects of the amyloid-cascade model almost uniformly failed to provide benefits in clinical trials, with some leading to worsening of symptoms and/or other adverse outcomes [6–9]. The unsettlingly consistent failure of clinical trials led to questioning of the amyloid cascade hypothesis, stimulating a search for alternative AD paradigms [10–13].

The task for a successful alternative is a formidable one, for it has to self-consistently accommodate most of the knowledge and data generated under the guidance of the amyloid cascade hypothesis within a parsimonious, testable, and predictive framework. Given the vastness and diversity of accumulated knowledge, the fragmentation of research by specialization and interdisciplinary barriers, and the limited capabilities of human memory and information processing, it may be worthwhile to explore a possibility of using computers in assisting experts to generate alternative viewpoints, hypotheses, and paradigms [14–16]. This is especially pertinent in the case of complex multifactorial diseases such as Alzheimer’s, where relevant information is distributed over a large number of disconnected expert domains.

Using algorithms for hypothesis independent search and expert-assisted synthesis of information, we attempted to parsimoniously reconcile multimodal clinical profiles of early-stage AD^*^ patients and accumulated research knowledge within a general conceptual framework.

In brief, a multimodal clinical profile of a patient (e.g., biochemistry, genetics, imaging, and medical records) is used by a human expert to generate a representative set of terms that characterize the disease configuration of the patient. The generated set serves as a query to search research literature and databases for information blocks (titles, abstracts, articles, and database entries) with highest densities of query terms. Selected and rank-ordered blocks of information are analyzed by an expert to identify a parsimonious set of concepts that interconnect cliques of search terms. The identified concepts are then used as new or additional query terms in the next iteration to identify higher level connectors, until all search terms become assimilated within a parsimoniously interconnected network. Analysis of the generated disease network by a human expert allows for formulation of de novo hypotheses. Although searches are non-exhaustive and hypothesis generation is inevitably biased by idiosyncratic expertise and choices of human expert, the relative worth of a generated hypothesis is measured in terms of its practical utility by testing hypothesis’ predictions empirically and/or in silico.

Results of our attempts to reconcile multimodal clinical profiles of early-stage AD patients with accumulated research information suggest that Alzheimer’s may not be a homogenous CNS disease, as traditionally assumed, but a heterogeneous, system-level, network disorder, which is driven by chronic network stress and dyshomeostasis. Since the latter can be incited by potentially diverse endogenous and exogenous factors and their interactions, AD may have potentially multiple etiologies and evolutionary trajectories that converge to a common clinicopathological endpoint recognized as Alzheimer’s disease.

In this report, we discuss biochemical and genetic evidence suggesting a central role of chronic tissue injury/dyshomeostasis, innate immune reactivity, and inflammation in the etiopathobiology of Alzheimer’s disease, and introduce a conceptual perspective on AD as a system-level network disorder. The implications of the proposed systemic nature of Alzheimer’s disease for treatment and prevention of cognitive decline are briefly discussed.

### Chronic inflammation and the reciprocity of central and peripheral disorders

Chronic low-grade inflammation, metabolic disorders (often of pro-diabetic type), endocrine dysregulation, and immune dyshomeostasis are common hallmarks shared by diverse chronic complex disorders, including Alzheimer’s disease. Over the last years, studies aimed at uncovering causes of these systemic dysfunctions revealed that chronic inflammation, whether central or peripheral, can be both a cause and a consequence of the progressive dysregulation of homeostatic controls and physiological systems in complex diseases [17–20].

Specifically, CNS neuroinflammation has been linked to the development and progression of metabolic, endocrine, and immune disorders, and their sequelae such as insulin resistance, hyperglycemia, dyslipidemia, metabolic syndrome, type 2 diabetes (T2D), autoimmune disorders, and cardiovascular and neurodegenerative diseases [21–24]. In pharmacological models of brain toxicity, a short-term brain stress inflicted by intracerebroventricular-injected thapsigargin, a chemical inducer of ER stress, was sufficient to induce glucose intolerance, systemic and hepatic insulin resistance, and blood pressure increase in laboratory animals. These systemic changes were accompanied by elevated sympathetic tone and prevented by sympathetic suppression, indicating mediation via the neural route [25]. Brain stress inflicted by intracerebroventricular-injected Aβ oligomers triggered peripheral glucose intolerance and other hallmarks of insulin resistance [26]. Engineered insulin resistance in the hippocampus, a major CNS target of AD, leads to unexpected systemic effects, including metabolic abnormalities, such as glucose intolerance, as well as anxiety and impaired cognition [27]. Aging animals in a murine AD model (3xTg-AD) develop a severe autoimmune/inflammatory disorder, accompanied by progressive systemic abnormalities and behavioral and cognitive deficits, which appear prior to significant β-amyloid or tau neuropathology [28]. In humans, hyperinsulinemic and hyperglycemic individuals show increased plasma and brain levels of β-amyloid [29–31], and earlier studies demonstrated peripheral glucose intolerance in AD patients [32]. By now, a link between Alzheimer’s disease and metabolic disorders has been firmly established, with patients with type 2 diabetes at increased risk of AD and vice versa [33].

At the same time, over the last years, it has been repeatedly demonstrated that peripheral immune challenges as diverse as skin and gastrointestinal inflammation, viral and microbial infections, toxic exposures, gut dysbiosis, and inflammation associated with atherosclerosis and obesity, can remotely elicit activation of innate immunity in the CNS, thereby driving or priming CNS neuroinflammation, which in turn can lead to peripheral disorders and AD-like neuropathology in the aging brain [12, 34–45]. Central and peripheral levels of inflammation have been linked to clinical outcomes in early-stage AD patients, and epidemiological studies suggest that elevation of inflammatory markers may be evident decades before the onset of clinical manifestations [46, 47]. The mechanisms by which the chronic inflammation and/or dysregulated immune responses associated with AD risk factors may influence individual susceptibility to neuroinflammation and AD neuropathology have been recently reviewed [48].

Altogether, accumulating evidence indicates that chronic CNS neuroinflammation and disorders can drive or contribute to chronic peripheral disorders and inflammation. At the same time, chronic peripheral immune challenges and inflammation can drive or contribute to CNS stress, neuroinflammation, and disorders. As central and peripheral abnormalities can potentially form circular loops that reinforce each other, a persistent driver(s) of tissue dyshomeostasis, whether central or peripheral, can potentially initiate and perpetuate chronic activation of innate immunity in the CNS and on the periphery, leading over time to progressive dysregulation of homeostatic controls, exhaustion and dysfunction of affected tissues and systems, and their diverse sequelae which we recognize as complex chronic disorders.

### Chronic stress response and tissue damage/dyshomeostasis in Alzheimer’s disease

Acute phase response (APR) is a centrally orchestrated reaction of the organism to stress or challenge. It is normally activated upon tissue injury, infections, stress, neoplasia, and inflammation. Historically associated with acute inflammation caused by pathogens, APR manifestations also accompany chronic inflammatory disorders [49–53].

Multimodal profiles of early-stage AD patients exhibit a number of remarkable similarities to a classical APR profile, which include hypozincemia, hypoferremia, hypercupremia, elevated cortisol, depressed thyroid hormone (T3) values, elevated complement (C3, C4), depressed steroid hormones, insulin resistance, hyperglycemic trends, negative nitrogen balance, loss of muscle mass, as well as depression, anxiety, and lethargy [52, 54–56].

**Figure 1:**
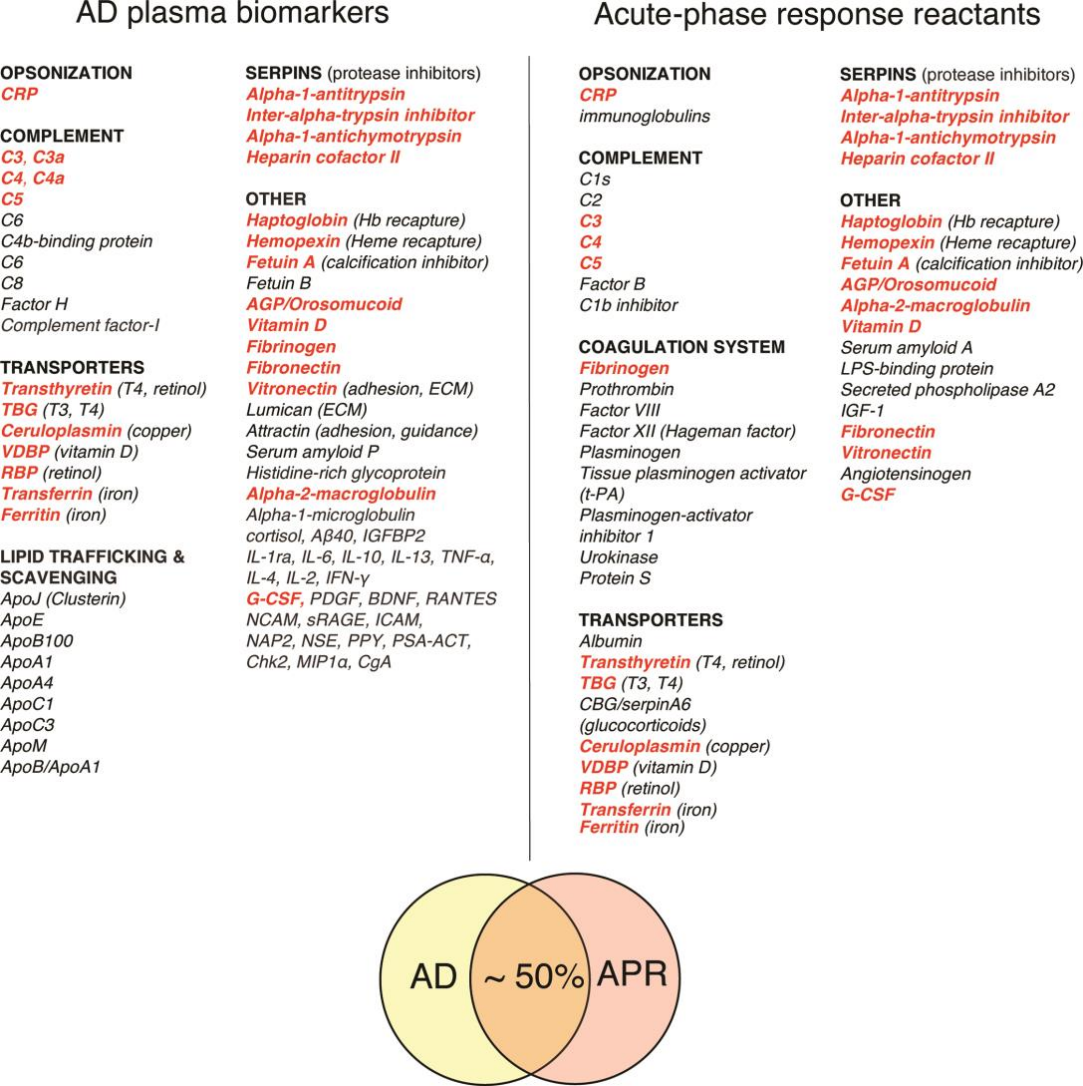
**Comparison of AD plasma biomarkers and classical acute-phase response reactants.** Shared factors are highlighted in bold red. Most AD biomarkers that are not classical acute phase response reactants represent either APR-related proteins or locally produced stress and/or signaling factors (see discussion in the text). Abbreviations: *CRP* (C-reactive protein); *TBG* (thyroxine-binding globulin); *CBG* (corticosteroid-binding globulin, alias transcortin, serpin A6); *VDBP* (vitamin D-binding protein); *RBP* (retinol-binding protein); *AGP* (alpha-1-acid glycoprotein, a.k.a. orosomucoid); *IGF-1* (insulin-like growth factor 1); *G-CSF* (granulocyte colony-stimulating factor); *Aβ40* (amyloid-beta 1-40); *IGFBP2* (insulin-like growth factor-binding protein 2); *IL-1ra* (interleukin 1 receptor antagonist; *IL-6, IL-10, IL-13, IL-4, IL-2* (interleukins 6, 10, 13, 4, and 2, correspondingly); *TNF-α* (tumor necrosis factor alpha); *IFN-γ* (interferon gamma); *PDGF* (platelet-derived growth factor); *BDNF* (brain-derived neurotrophic factor); *RANTES* (regulated on activation normal T cell expressed and secreted, a.k.a. CCL5, chemokine (C-C motif) ligand 5); *NCAM* (neural cell adhesion molecule); *sRAGE* (soluble receptor for advanced glycation end-products); *ICAM* (intercellular adhesion molecule); *NAP2* (nucleosome assembly protein 2); *NSE* (neuron-specific enolase); *PPY* (pancreatic polypeptide); *PSA-ACT* (prostate-specific antigen-alpha-1-chymotrypsin complex); *Chk2* (serine/threonine-protein kinase Chk2); *MIP1α* (macrophage inhibitory protein 1-alpha); *CgA* (chromogranin A); *ApoJ (Clusterin)*, *ApoE*, *ApoB100*, *ApoA1*, *ApoA4*, *ApoC1*, *ApoC3*, *ApoM*, and *ApoB* (apolipoproteins J, E, B100, A1, A4, C1, C3, M, correspondingly). *Compilation references*: acute-phase response reactants [49, 52, 53, 57, 58]; AD plasma biomarkers [59–63].

Figure 1 compares a representative list of classical acute phase response reactants (APRRs) that are secreted by the liver into systemic circulation during the acute phase of stress response and a representative list of promising AD plasma biomarkers compiled from biomarker discovery studies [49, 52, 53, 57–63]. A conservative estimate of the overlap between APRRs and AD biomarkers is approximately 50%. Moreover, a closer inspection of AD plasma biomarkers that are not on the list of systemic APRRs reveals that virtually all of them are either factors related to systemic APRRs or products of local stress responses. The latter group of molecules include various scavengers, transporters, chaperons, adhesion and guidance factors, cytokines, chemokines, growth factors, complement components, and elements of extracellular matrix (ECM). These factors are produced and secreted in damaged territories by activated resident and recruited cells and surrounding tissue in response to tissue stress, damage, inflammation, and infection. Locally secreted stress factors perform diverse functions, such as promoting and resolving inflammatory reactions, promoting vascular and tissue permeability, dismantling, remodeling, and restoring ECM, scavenging spilled materials, and assisting in wound clearance, tissue repair, and repopulation processes. C4a, TGF- β1, and MMP-9 – plasma biomarkers that are characteristically upregulated in the chronic inflammatory response syndrome (CIRS) and many AD patients – are examples of stress factors produced in damaged and inflamed territories [54, 64–67].

A comparative analysis of classical APRRs and AD plasma biomarkers may provide valuable insights into the nature and targets of AD-associated tissue damage and inflammation. In this regard, the most evident differences between the lists shown in Figure 1 are a large number of apolipoproteins and locally produced stress factors among AD plasma biomarkers, which are virtually absent from the list of systemic APRRs.

Both in normalcy and in disease, apolipoproteins mediate the export, import, trafficking, and redistribution of lipophilic species, including lipids, cholesterol, and lipophilic waste and toxins, both within and across multiple levels of organizational hierarchy, from cells to the whole organism [68–70]. Correspondingly, apolipoproteins function as scavengers of lipophilic species, ligands for cell-surface receptors, and as essential structural components of lipoprotein particles that transport lipophilic species in blood and other body fluids.

The apparent abundance of apolipoproteins among promising AD biomarkers may indicate chronic tissue injury, oxidative stress and damage of lipid membranes, dysregulation of cholesterol/lipid metabolism or trafficking, active detoxification, and/or a shift to tissue-independent cellular phenotypes and metabolic modes that are associated with excessive production and export of lipids and cholesterol. The abundance of apolipoproteins among potential AD biomarkers is well consistent with typical lipid profiles of early-stage AD patients, which often show signs of dyslipidemia and hypercholesterolemia, including high levels of total cholesterol, low-density lipoprotein cholesterol (LDL-C) and, not infrequently, high-density lipoprotein cholesterol (HDL-C) (not shown).

Altogether, biochemical profiles of AD patients, whether they are analyzed on a case-by-case basis (clinical data) or by averaging over patients’ populations (biomarker discovery studies), suggest that chronic tissue damage/dyshomeostasis and ongoing local and/or systemic stress responses may represent characteristic features shared by many AD patients. Since the steady-state plasma levels of locally produced stress factors are chronically and often substantially elevated in a large fraction of AD patients, it is reasonable to suspect that chronic tissue injury/dyshomeostasis and inflammation in these individuals affect relatively large surface areas, such as that of lungs, intestines, vasculature, and/or lymphatics.

### Genetics of Alzheimer’s disease

**Figure 2:**
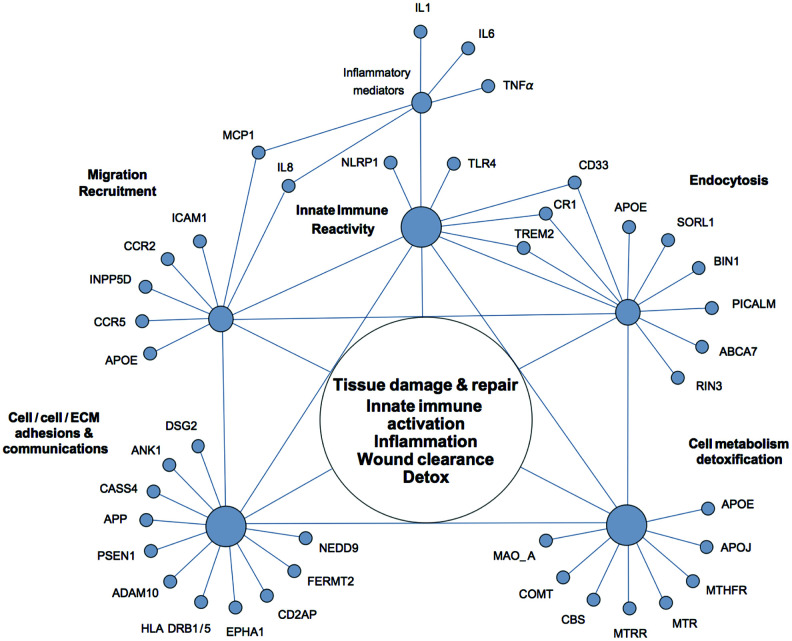
**The network of genetic polymorphisms associated with Alzheimer’s disease.** Most of the genetic polymorphisms associated with Alzheimer’s disease can be broadly assigned to a small number of overlapping functional groups and interconnected via such terms as tissue damage and repair, innate immune activation, inflammation, wound clearance, and detoxification, without specifying whether these processes occur in the CNS or on the periphery.

Alzheimer’s disease is a multifactorial neurodegenerative disorder with a strong genetic component and a high degree of heritability. Over many years of research, multiple and remarkably diverse genetic risk factors have been identified in association with AD [71–73]. However, attempts to rationalize discovered associations from the perspective of the amyloid cascade hypothesis of Alzheimer’s disease have thus far fallen short of unifying genetic AD susceptibility factors within a general, self-consistent framework. Although a number of authors noted that unexpectedly many AD-associated polymorphisms occur in proteins with functions in innate immunity, such associations are typically interpreted in terms of inappropriate CNS neuroinflammation and β-amyloid generation and/or clearance [73–76]. We suggest that, by relaxing the assumption that AD is solely a disease of the brain, the majority of genetic AD susceptibility factors can be parsimoniously integrated via such terms as chronic tissue damage/dyshomeostasis, innate immune reactivity, inflammation, and ongoing wound clearance and tissue repair, without specifying whether these processes occur in the CNS or on the periphery (Figure 2).

Indeed, most of AD susceptibility factors can be conditionally assigned to a small number of overlapping functional groups. The largest group comprises proteins that are involved in the regulation of innate immune reactivity and inflammatory responses. AD risk is associated with genetic polymorphisms in several key sensors of innate immunity such as NLRP1 (NACHT, LRR and PYD domains-containing protein 1) and TLR4 (toll-like receptor 4), and over a dozen of immune mediators, which include pro-inflammatory cytokines IL-1, TNF-alpha, and IL-6, adhesion molecules such as ICAM-1 (intercellular adhesion molecule 1), chemokines IL-8 (interleukin 8) and MCP-1 (monocyte chemotactic protein 1), and chemokine receptors, such as CCR2 and CCR5 [75, 77]. Many of the AD susceptibility factors identified in large-scale genome-wide association studies (GWAS) also have functions in innate immunity and inflammatory responses [74, 78]. These include CD33 (Siglec-3) (a myeloid cell-surface receptor), TREM2 (triggering receptor expressed on myeloid cells 2), MS4A6A and MS4A4AE (membrane-spanning proteins expressed on myeloid cells), CR1 (complement receptor 1), INPP5D (SHIP-1) (phosphatidylinositol (PtdIns) phosphatase), ApoE4 (apolipoprotein E epsilon 4 allele), SORL1 (sortilin-related receptor L), ABCA7 (ATP-binding cassette sub-family A member 7), PICALM (phosphatidylinositol-binding clathrin assembly protein), BIN1 (bridging integrator protein 1), and RIN3 (Ras and Rab interactor 3). Functional descriptions and disease associations of these proteins can be found in the Appendix, Table 1, and elsewhere [71–73].

**Table 1:** A quick reference guide for select AD susceptibility factors.

**Name**	**Major functions**	**Expression**	**Disease associations**
**Innate immune reactivity and inflammatory response**			
**NLRP1**	A sensor of intracellular PAMPs and DAMPs; the sensor component of the NLRP1 inflammasome; triggers inflammation in response to microbial products, particulates, crystals (e.g., cholesterol and urate crystals), silica, asbestos, Aβ, prions, mutant SOD1, etc.	Broad; abundant in immune and epithelial cells	AD, chronic peripheral inflammatory and autoimmune disorders
**TLR4**	A major sensor of extracellular PAMPs and DAMPs; triggers inflammation upon recognition of various ligands, including LPS, viruses, bacteria, oxLDLs, saturated fatty acids, heat shock proteins, Aβ, fibronectin, fetuin-A, and β-defensins	Predominantly in cells of myeloid origins	AD, macular degeneration, Crohn’s disease, ulcerative colitis, tonsillitis, infections
**CD33**	A receptor for sialic-acid SAMPs (self-associated molecular patterns); functions in cell-cell interactions, endocytosis; suppresses activation of immune cells when engaged with cognate SAMPs	Predominantly in innate immune cells	AD
**TREM2**	A cell-surface receptor; involved in initiation and suppression of inflammatory responses in innate immune cells; functions in phagocytosis	Broad; augmented in brain, lung, and adipose tissues	AD, COPD, GI injury, infections, Nasu-Hakola disease
**CR1**	A cell surface immune adherence receptor (complement system); phagocytosis of cells, debris, and pathogens opsonized by complement factors, e.g., C1q, C3b, and C4b	Erythrocytes, monocytes, leucocytes, neutrophils, but not in brain cells	AD, chronic peripheral inflammatory and autoimmune disorders, infections
**INPP5D**	A phosphatidylinositol phosphatase; involved in signaling of cell surface receptors; regulates multiple functions in immune cells, including chemotaxis, activation, homeostasis, and phagocytosis	High in immune cells, bone marrow, lymphoid tissues	AD
**RIN3**	A Ras effector and Rab5-directed guanine nucleotide exchange factor; cell signaling; endocytosis, migration, synaptic functions, immune responses	Broad; abundant in mast cells	AD, Paget’s disease
**PICALM**	Phosphatidylinositol-binding clathrin assembly protein; endocytosis	Broad	AD, acute leukemias
**Cell-cell and cell-matrix adhesions and communications**			
**DSG2**	Desmosomal cadherin, an integral component of intercellular junctions; mediates functional cell-cell adhesions; links plaque proteins and cytoskeleton; regulates EMT and barrier functions	High in epithelial cells, cardiomyocytes, cancer	AD, arrhythmogenic right ventricular dysplasia, cancer
**ANK-1**	Ankyrins link membrane proteins and adhesion molecules to cytoskeleton; involved in integrating cells into tissues and regulation of cell motility, activation, proliferation, and EMT	RBCs, bone marrow, lymphoid tissue, brain	AD, spherocytosis, hereditary hemolytic anemia
**NEDD9**	A scaffold of the Cas protein family with functions in cell adhesions, cell attachment, migration, and invasion; regulates EMT; metastatic marker in multiple cancers	Broad	AD, cancer, hemochromatosis
**CASS4**	A scaffold of the Cas family of proteins; functions in cell attachment, migration, and motility via regulation of focal adhesion kinases in focal adhesions	Abundant in lungs, spleen, and leucocytes	AD, atopic asthma, cystic fibrosis, lung cancer
**CD2AP**	A scaffolding molecule that mediate attachment of cell surface receptors to the cytoskeleton; functions in the formation of adherens junctions and EMT; involved in maintaining integrity and permeability of barriers, including BBB, via control of adherens junctions	Broad; low in brain; high in kidneys, endothelial and epithelial cells	AD, renal disease
**FERMT2**	A member of the fermitin family of focal adhesion proteins; involved in integrin activation, integrin signaling, and cell adhesion; functions in adherens junctions and plays a role in wound healing, tissue repair, angiogenesis; overexpressed in cancers where it promotes EMT and invasion	Broad; high in endothelial cells	AD, cancer
**EPHA1**	A member of the cell surface ephrin receptor family that mediate contact-dependent bidirectional cell-cell communications. Eph receptors are involved in shaping tissues during development and injury, and support functions and homeostasis of mature tissues; upregulated at sites of injury and inflammation; involved in the control of endothelial, blood-brain, and intestinal barrier permeability, EMT, neural development, plasticity, and regeneration; overexpressed in carcinomas	Broad	AD, cancer, chronic inflammatory disorders
**APP**	A cell surface receptor with functions in axonal guidance, neuronal adhesions, and synaptogenesis; regulates neurite outgrowth through binding to ECM components (heparin and collagens); signals to nucleus via conserved YAP and TAZ transcription factors that control EMT	Moderate in the brain, low in GI and other epithelia	AD, cancer
**ADAM10**	A broad specificity membrane metalloprotease that cleaves and sheds extracellular domains of transmembrane proteins; a key α-protease in APP processing; regulates cell-cell adhesions, migration, communications; may function in EMT; substrates: Notch, APP, N-/E-cadherins, Klotho, VEGF, TNF-α and IL6 receptors, ephrins, and many other molecules	Broad, widely in most immune cells	AD, breast cancer
**Presenilin-1**	A key component of the γ-secretase complex that catalyzes intramembrane cleavage of integral membrane proteins; involved in regulation of cell adhesions, fate, migration, neurite outgrowth, synaptogenesis; substrates: Notch, APP, DSG2, N-/E-cadherins, SORL1, LRP1, Klotho, HLA; VEGF, IL1 and IL6 receptors, ephrins, insulin receptor, and many other molecules	Broad, high in cerebral cortex, thyroid gland, respiratory and GI systems	AD, FTD, Pick’s disease, cardiomyopathy, cancer
**Cell metabolism and detoxification**			
**MTHFR, MTR, MTRR, CBS**	Enzymes of folate-dependent one-carbon metabolism (OCM), a central hub of the basic cellular metabolism; OCM supports a large variety of metabolic pathways and reactions utilized in biosynthesis of proteins, lipids, nucleic acids, and neurotransmitters. OCM is essential for tissue and cell repair, cell proliferation, DNA repair, detoxification, and antioxidant defenses	MTHFR, MTR, MTRR- broad; CBS - predominantly in liver, hepatocytes, and CNS	AD, and neurological, psychiatric metabolic, cardiovascular, immune and hematological disorders; homocystinuria, cancer
**ApoE**	A multifunctional apolipoprotein with a best-known role in lipid metabolism, and trafficking and redistribution of lipids and cholesterol within and between cells and tissues, particularly in the brain; functions as a stress factor (chaperon/scavenger) secreted upon injury; Aβ chaperon	High in the brain, liver, adrenals, low to moderate elsewhere	AD, FTD, Pick’s disease, TBI, atherosclerosis, coronary heart disease, infections
**ApoJ**	A member of the small heat shock protein family; a generic stress factor (chaperon/scavenger); upregulated and secreted upon cell stress/injury; functions in lipid metabolism/transport, cell adhesion, apoptosis; ApoJ and ApoE are primary chaperons in Aβ clearance from the brain	Broad	AD, HD, atherosclerosis, cancer, cardiovascular and metabolic disorders
**ABCA7**	An ATP-binding cassette transporter; functions in lipid transport and homeostasis (predominantly in immune cells), and macrophage-mediated phagocytosis	High in leukocytes, bone marrow, thymus, spleen	AD
**SORL1**	A multifunctional endocytic, transport, and sorting receptor; mediates uptake of lipoproteins and proteases; involved in APP trafficking and sorting	High in the brain, low to moderate elsewhere	AD, vascular disease
**COMT**	Methylase; targets reactive catechol compounds for degradation and clearance;substrates: epinephrine, norepinephrine, catechol-estrogens, drugs, other compounds	Broad	AD, impaired cognition, behavioral and psychiatric disorders
**MAO-A**	Monoamine oxidase; oxidative deamination of monoamines; substrates: serotonin, melatonin, dopamine, epinephrine, norepinephrine, other compounds	Broad	AD, neurological and psychiatric disorders

For details, references, abbreviations, and additional risk factors, see Appendix, Figure 2, and discussion in the text.

It is worthy of note that a recent pathway analysis of AD susceptibility factors indicates that APOE, INPP5D, TREM2, ABCA7, CR1, PICALM, and BIN1 are jointly involved in three functional categories – immune responses, movement of phagocytes and myeloid cells, and engulfment of extracellular material – i.e., in the fundamental innate immune processes that mediate tissue homeostasis, wound clearance, and tissue repair [73].

Another major group of AD susceptibility factors include proteins with functions in cell-cell and cell-matrix contacts and adhesions, where they mediate communications between the extracellular environment and the cell interior. Most proteins from this group play major roles in reversible transitions between quiescent and activated cellular states and phenotypes. Examples of such transitions include EMT (epithelial-to-mesenchymal transition) and its opposite, MET (mesenchymal-to-epithelial transition) [79, 80], endothelial-mesenchymal transitions (EndMT) [81], loosening and tightening of permeability barriers, and activation and deactivation of immune cells.

In the context of organized tissues, a breakdown or loosening of cell-cell and cell-ECM connectivity, e.g., due to tissue injury or dyshomeostasis, leads to cell activation and transition to partially dedifferentiated, motile, endocytic, and pro-secretory cellular phenotypes, which allow for tissue/anchorage-independent activity, survival, and proliferation/expansion. The reverse process involves the (re)establishment of stable cell-cell and cell-ECM connectivity, which inhibits cell activation and independent cellular activities, leading to quiescent, tissue-dependent, and well-differentiated cellular phenotypes. Cellular activation and transition to individualized phenotypes is characteristically accompanied by step-wise mode-switching in cellular metabolism from oxidative phosphorylation to less efficient but more flexible, (glyco)lytic modes of cellular metabolism, which are associated with cell proliferation, migration, and expansion. The reverse shift from (glyco)lytic metabolism to oxidative phosphorylation occurs upon restoration of intercellular connectivity, anchorage, and formation of organized multicellular assemblies, networks, and tissues [80–83].

Examples of AD susceptibility factors that belong to this group include DSG2 (Desmoglein-2), ANK-1 (Ankyrin-1), NEDD9 (Neural Precursor cell expressed Developmentally Downregulated 9), CASS4 (Cas scaffolding protein family member 4), PTK2B (Protein-tyrosine kinase 2-beta / Focal adhesion kinase 2), CD2AP (CD2-associated protein), FERMT2 (fermitin family homolog 2), APP (amyloid precursor protein), PSEN1 (Presenilin-1), ADAM10 (a disintegrin and metalloproteinase 10), EPHA1 (Ephrin type-A receptor 1), TREM1, TREM2, CD33, and HLA-DRB1/HLA-DRB5 (Major histocompatibility complex class II, DR beta 1/5) (see the Appendix and Table 1 for functional descriptions).

Notably, many proteins from this group are abundantly expressed and have important functions in epithelial, endothelial, and immune cells. Several proteins (e.g., DSG2, CD2AP, EPHA-1) are involved in the regulation of permeability barriers.

Chronic inflammation and transition to tissue-independent, individualized cellular phenotypes and (glyco)lytic metabolism are hallmarks of cancer. Perhaps not coincidentally, a number of AD susceptibility factors in this group (e.g., NEDD9, CASS4, and PTK2B) are cancer signaling proteins [84]. NEDD9 (a.k.a. CASS2), for example, is a positive regulator of EMT and a metastasis marker in multiple cancers [85].

The third group of genetic AD risk factors comprises proteins with functions in metabolic pathways that are critical for proliferation, maintenance, and repair of cells and tissues, as well as for detoxification and antioxidant defenses. These include i) key enzymes of one-carbon metabolism, such as MTHFR (methylenetetrahydrofolate reductase), MTR (5-methyltetrahydrofolate-homocysteine methyltransferase, a.k.a. methionine synthase), MTRR (5-methyltetrahydrofolate-homocysteine methyltransferase reductase, a.k.a. methionine synthase reductase), and CBS (cystathionine β-synthase); ii) proteins involved in lipid transport and metabolism (ApoE, ApoJ, ABCA7, and SORL1); and iii) detoxifying enzymes such as COMT (catechol-O-methytransferase) and MAO-A (monoamine oxidase A), which catabolize reactive catechol and amine compounds, correspondingly. Brief discussion of these proteins can be found in the Appendix and Table 1.

Altogether, AD genetic risk factors provide few indications that Alzheimer’s disease is solely or even largely a brain disease. Instead, AD genetics appears to center squarely on innate immune reactivity, cell-cell and cell-matrix connectivity and communications, and housekeeping metabolism, i.e., on the processes and systems that are essential for maintaining multicellular organization and tissue homeostasis, whether in the CNS or on the periphery. Moreover, AD genetics appears to suggest that AD risk is associated with enhanced innate immune reactivity and/or pliability of cellular connections, the traits that are advantageous in young individuals living in volatile environments with diverse and varying acute insults. However, enhanced innate immune reactivity and/or pliability of cellular connectivity can be detrimental in such conditions as chronic tissue stress or dyshomeostasis, non-resolving inflammation, and aging, leading to exaggerated tissue damage, progressive disconnectivity of cellular networks, immune dysregulation, and the type of low-level inflammation that accompanies diverse chronic complex conditions, including neurodegenerative disorders [17, 86]. In terms of cell types and systems that are preferentially affected by AD-associated polymorphisms, innate immunity emerges as an apparent leader, which is closely followed by epithelial, endothelial, and neuronal cells, and their organized assemblies, such as epithelial and endothelial barriers and neuronal networks.

Overall, AD plasma biomarkers and genetics are in a marked agreement one with another, both suggesting the central role of chronic tissue injury/dyshomeostasis, innate immune reactivity, and inflammation in the AD etiopathobiology. However, due to potential diversity of factors and conditions that can drive chronic tissue stress and dyshomeostasis, whether on the periphery or in the CNS, population-averaged data may mask diversity of the gene-environment interactions and mechanisms that drive cognitive decline in individual patients, necessitating individualized approaches to both diagnostics and therapy.

Indeed, metabolic profiling of early-stage AD patients on the individual basis allows for stratifying patients into several subtypes, which are responsive to subtype-tailored therapeutic regimens [54, 55, 64]. Likewise, a preliminary analysis of AD genetic profiles on the one-by-one basis suggests that individual genetic predispositions tend to come as specific combinations rather than as random sets (unpublished observations). The latter point may be important because most AD-associated genetic polymorphisms are common and provide only marginal contributions to AD risk, with typical odds ratios between 1.1 and 1.5, notwithstanding few exceptions such as APOE4 and TREM2. However, multiple polymorphisms affecting the same pathway or functionally interrelated processes in a synergistic manner can create “weak spots” in individual make-ups, increasing the disease risk multifold. When, in a person, a genetic “weak spot” is chronically challenged by environmental factors, infection, or a disease, this may make all the difference for the person, but be largely irrelevant for others who share the same environment but carry unrelated polymorphisms.

### Shifting perspective: from an organ to the system

The conventional model of AD pathogenesis begins with β-amyloidosis and is followed by neuroinflammation and tauopathy. However, multiple drug candidates that target β-amyloidosis uniformly failed to confer benefits in clinical trials, and some led to adverse outcomes, although in a number of cases reduction in β-amyloid burden could be demonstrated. Targeting neuroinflammation with inhibitors of microglial activation yielded no success either [6–9]. One possible explanation of these unexpected outcomes is that β-amyloidosis and neuroinflammation are consequences of a non-resolving protective response of brain tissue to various chronic brain perturbations, which can potentially have diverse origins, central and/or peripheral.

Indeed, expression of the Alzheimer’s amyloid precursor protein (APP) gene is regulated by acute phase reactants and proinflammatory cytokines, and the levels of APP and its metabolite Aβ increase rapidly following chemical or traumatic injury, infections, or exposure to volatile toxins and air pollution [12, 87, 88]. These observations, together with the recently discovered protective properties of Aβ, such as its potent antimicrobial and antiviral activity, scavenging and sequestration of pathogens and metals, and antioxidative and neuroprotective activities, suggest that Aβ may be an innate immune effector, whereas Aβ generation is a normal physiological response to various brain stresses [11, 55, 89–92]. From this perspective, innate immune reactivity and Aβ production in the brain are seen as a double-edge sword. Beneficial in the context of normal brain physiology and positive stress, innate immune responses and their effectors can become harmful in conditions of non-resolving CNS distress and inflammation. In fact, authors of the amyloid cascade hypothesis recognized that “there may be many causes of Alzheimer’s disease” but postulated “that APP mismetabolism and β-amyloid deposition are the primary events in the disease process” [2]. However, targeting effectors and downstream consequences of a protective response without first identifying and addressing the primary causes of chronic CNS stress and neuroinflammation may be a counterproductive or ineffective therapeutic strategy.

Significantly, the drivers of or contributors to chronic CNS distress and inflammation may or may not be located in the brain, as accumulating research evidence demonstrates that diverse peripheral stresses and immune challenges, including gastrointestinal inflammation, dysbiosis, infections, dietary and inhalational toxicity, and inflammation in tissues affected by chronic disease, can remotely prime, drive, or contribute to CNS stress and neuroinflammation via neural, immunological, and humoral routes [12, 35–38, 93]. Reciprocally, altered brain outputs via the hypothalamic-pituitary-adrenal (HPA) axis and the autonomic nervous system (ANS) influence peripheral metabolism [21], respiratory and gastrointestinal functions [94], intestinal barrier permeability [95], composition of gut microbiota [96], virulence of pathogens [97], and immune responses and inflammation in peripheral tissues [98, 99]. As a consequence of the central network position of the CNS in the systems connectomics, chronic peripheral immune challenges, toxicity, and inflammation are communicated to the CNS, where they can drive or contribute to stress, dyshomeostasis, and inflammation. Conversely, chronic stress, neuroinflammation, lesions, maladaptive alterations, and neurodegeneration in the brain, particularly when they target key CNS circuits responsible for homeostatic controls, can drive or contribute to dyshomeostasis and inflammation on the periphery, which in turn can feed back to further brain stress, inflammation, and disorder. Hence, endemic “chicken-and-egg” situations that commonly arise when one attempts to interpret associations between AD and its diverse comorbidities using a linear cause-effect logic.

The emerging picture may not be surprising, since the brain is not an isolated organ but an integral part of a complex dynamic network of the interconnected and interdependent cells, tissues, and physiological systems that constitute the organism. The brain is a central hub of the nervous system, which receives inputs from and sends outputs to virtually all tissues and organs to dynamically regulate and coordinate their functions and homeostasis. The brain and the nervous system are the principal sensors, integrators, processors, and effectors of external and internal stresses, and thereby their major targets.

Moreover, breakthrough discoveries at the intersection of neuroscience and immunology have revealed that the nervous system and the immune system are functionally and structurally intertwined, both in the CNS and on the periphery, performing essentially as a global sentinel system that senses, integrates, evaluates, and responds to stimuli that threaten or are perceived to threaten homeostasis [100]. Long-range neural transduction and bidirectional neuroimmune communications between neuronal and immune cells interlink central and peripheral innate immunity, and emerge as potential mediators of reciprocal relationships between peripheral and central inflammation and disorders [34, 98, 99, 101].

Significantly, upon impact of excessively acute or prolonged stress, neuronal and/or immune components of the neuroimmune sentinel system can acquire long-lasting functional and/or structural alterations in the CNS and/or on the periphery. This creates a potential for the establishment of neurogenic and/or immunogenic hypersensitivity and inflammation, and the emergence of circular loops that link and mutually reinforce peripheral and central abnormalities and inflammation. For example, repeated exposure to respiratory allergens induces lasting functional alterations in the sensory airway neural pathway, which involve neuroplastic changes in the peripheral afferent airway nerves, and neuronal and glial sensitization and gliosis in the brainstem nuclei. These acquired alterations are thought to cause the airway hyperresponsiveness, a characteristic feature of asthma [102]. Similar neuroimmune alterations are proposed to underlie “cough hypersensitivity syndrome” [103]. Neurogenic inflammation triggered by chemical exposures is hypothesized to play a role in the multiple chemical sensitivity syndrome and the chronic inflammatory response syndrome [100, 104].

On the side of innate immunity, microbial or sterile immune challenges can lead to long-term functional alterations in innate immune cells (“trained immunity”), possibly via epigenetic and metabolic reprogramming, which result in either enhanced or suppressed immune responses to subsequent stimuli [105, 106]. Accumulating evidence implicates both peripheral and central innate immune memories and their cross-talk in AD pathogenesis [107]. For example, experiments in an animal model of AD demonstrate that peripheral inflammatory stimuli can induce long-term innate immune memories in the brain that influence neuropathology later in life [108]. A CNS variant of trained immunity is microglial priming, which recently emerged as a major contributor to a pro-inflammatory CNS milieu associated with the aging brain and CNS disorders, including AD, PD, MS, and traumatic brain injury [35, 109, 110]. The process of priming involves activation and proliferation of stimulated cells, and transition to a state of enhanced responsiveness, leading to exaggerated inflammatory reactions to subsequent inflammatory stimuli. Priming is a generic phenomenon that occurs in the CNS and on the periphery in many long-lived immune cells, including microglia, macrophages, mast cells, memory T cells, and natural killer cells.

Biological homeostasis is a state of minimum energy dissipation maintained by a dynamic, yin-yang balance of counteracting forces. When forces are imbalanced, homeostasis is challenged and stress ensues. Stress response is activated to mobilize and to redistribute local, regional, and systemic energy and resources to cope with stress in a most efficient manner and to promptly return the system to a balanced, homeostatic state of minimum energy dissipation [56, 94, 111].

Unlike acute stress, chronic stress makes a biological system to dwell for long periods of time in imbalanced, dissipative states of activation. As a result, activated cells, tissues, and systems incur and accumulate over time metabolic deficits, which they initially compensate by drawing on local and systemic reserves, thanks to facile redistribution of energy and resources at the network level [56]. However, in conditions of non-resolving stress, chronically activated cells, tissues, and systems sooner or later enter the exhaustion phase, which can lead to further network imbalances and stress that ramify throughout the systems network structure, affecting other domains and the system as a whole. As the systems network attempts to compensate for and minimize network stress and imbalances by rewiring functional and structural interrelations, it may become trapped in misconfigured states of chronic, dissipative dyshomeostasis, locally and/or globally, not unlike a misfolded protein.

AD patients present with diverse and multiple signs of dyshomeostasis and/or exhaustion in network domains involved in stress response, immune responses, anti-oxidant defense, house-keeping metabolism, and neuronal functions and support. Examples include depressed steroid hormones, altered thyroid function, signs of adrenal fatigue, decreased neurotrophic support (e.g., BDNF, GDNF, NGF, and irisin) [112, 113], insulin resistance, altered lipid and glucose metabolism, elevated homocysteine levels, negative nitrogen balance, and low systemic levels of essential micronutrients, vitamins, and enzymatic co-factors, such as B-complex vitamins (B6, B9 (folate), and B12), vitamin A, vitamin E, vitamin C, vitamin D (25-hydroxycholecalciferol), zinc, S-adenosylmethionine (SAM), and docosahexaenoic acid (DHA) [64, 114–116]. Peripheral monocytes of AD patients exhibit a decreased capacity for phagocytosis, which can be restored by targeted metabolic supplementation, both *ex vivo* and in patients [117, 118]. The plasma levels of major neuroendocrine inhibitors of innate immune reactivity, such as acetylcholine, cortisol, vasoactive intestinal peptide (VIP), melanocyte-stimulating hormone (α-MSH), estrogen, and norepinephrine, are typically altered in AD patients, suggesting either chronic hyperactivity or, more often, exhaustion of neural controls of immune reactivity [54, 64, 119].

Epidemiological surveys have identified diverse risk factors for AD and cognitive decline that have peripheral origins. These include vascular disease, diabetes, chronic infections, systemic inflammation, obesity, midlife chronic obstructive pulmonary disease and asthma, chronic periodontitis, midlife rheumatoid arthritis, head trauma, and reduced physical activity [12, 13, 120–122]. In addition, major contributors to brain stress and cognitive decline may also include insulin resistance, chronic hyperglycemia, obstructive sleep apnea, dietary sensitivities, gastrointestinal hyperpermeability, dysbiosis, and exposure to environmental toxins such as metals, chemicals, air pollution, volatile organic compounds, and biotoxins [12, 116, 123, 124]. The major themes that unite the vast majority of these diverse potential contributors to cognitive decline are chronic tissue injury or dyshomeostasis, chronic or recurrent stimulation of innate immunity, low-grade inflammation, and impaired homeostatic controls. As discussed above, essentially the same themes unify genetic risk factors for Alzheimer’s disease and are clearly reflected in biochemical and clinical profiles of AD patients.

Altogether, it is clear that the systemic context of the brain is more important than the conventional AD paradigm implies by focusing attention of researchers on the mechanisms and consequences of β-amyloidosis. Although the systemic context of each individual brain is different, the topological organization of the systems network that constitutes the human organism is universal. As a consequence of its central network position, the brain is a preferential target of stress, toxicity, and perturbations propagating through the systems network, whatever their origins may be, central or peripheral, external or internal. Depending on the entry portals and natures of chronic stress, toxicity, and inflammation, and the route by which they preferentially access the CNS, different CNS hubs and circuits can be targeted, potentially giving rise to different neurological disorders and clinical phenotypes. By virtue of their key network positions and functions, CNS hubs and neural systems of high network centrality (e.g., the stress system) are likely to be affected in many different CNS disorders. Hence, diverse yet overlapping neurological disorders and diverse yet overlapping clinical phenotypes within a given disorder. Hence, the potential heterogeneity of etiologies and evolutionary trajectories that converge to a common yet heterogeneous clinicopathological endpoint recognized as AD, PD, or MS.

To conclude, we have attempted to illustrate how a large number of diverse AD-related phenomena can be understood within the systems network perspective on Alzheimer’s disease. The outlined framework is not intended to be exhaustive but presented only as a way of organizing thinking and understanding of the processes that drive AD and possibly other neurological disorders. The framework is compatible with the amyloid cascade hypothesis and with most alternative AD hypotheses, which typically implicate diverse external and internal factors (e.g., infections, toxic metals, chemical exposures, ammonia, and cholinergic decline) as potential causes of AD. Clearly, the studies cited only begin to address the themes and ideas outlined above, and more systematic systems-scale research is needed. In many instances, the presented perspective leads to expectations and recommendations that are discordant with the currently dominant literature. Nevertheless, we would like to suggest that the ideas outlined herein are likely to be of higher pragmatic value in regard to diagnosis, prevention, and treatment of AD, and more descriptive of the actual factors and processes that drive the initiation and progression of AD and other complex neurological disorders.

### Clinical evidence and ramifications

Effective treatment of Alzheimer’s disease has been lacking and multiple clinical trials of monotherapeutics targeting various aspects of the conventional AD model have been consistently unconvincing. As an alternative, one of the authors (DEB) introduced recently a system-level programmatic approach for treating early stage AD, which showed promising results in case studies, including sustained arrest and reversal of cognitive decline [116, 125]. Rather than assuming a single cause and a single, linear mechanism of the disease, we opted for systematically evaluating the many potential contributors to cognitive decline in each individual patient and addressing them in a comprehensive, system-wide manner. Because each patient has a different combination of potential contributors and their weights, the approach to treatment is targeted and personalized [55, 64].

In brief, multimodal diagnostic tools, such as biochemical tests, genetic analysis, functional brain imaging, brain volumetric analysis, neuropsychological assessment, and evaluation of medical records and life histories, are used to determine major potential contributors to cognitive decline and network imbalances for each individual patient. On the basis of individual evaluation, a multimodal personalized therapeutic program is designed and applied to address the identified contributors and imbalances. The program involves multiple therapeutic modalities, which can include pharmacological, nutritional, and life style interventions, targeted dietary supplementation, hormonal optimization, probiotics, fasting, physical exercise, sleep optimization, stress management, meditation, and brain training [116, 125]. Multiple therapeutic modalities are aimed at synergistically impacting multiple domains and hierarchical levels of the systems network to maximize therapeutic effect. The major focus is on metabolic support and optimization of neuronal and other vital network functions. The approach to treatment is streamlined by stratifying patients in a number of AD subtypes and applying subtype-tailored therapeutic regimens [64].

As a proof of principle, a recent report describes 100 early-stage AD patients treated with the programmatic approach, who show documented improvement in cognitive performance and, in a number of cases, documented improvement in electrophysiology or imaging [55]. Given that the successful results were obtained by several different physicians at multiple sites across the country, the approach appears to be reproducible, scalable, and practicable by many physicians. Most importantly, improvements are typically sustained, unless the protocol is discontinued, suggesting that the root causes of the pathophysiological process are affected. As a general rule, patients in the earliest stages of cognitive decline respond more readily and completely than those with more advanced illness, demonstrating the overwhelming importance of early diagnosis and prevention of cognitive decline.

Certainly, the numbers of successful case studies and the unprecedented outcomes obtained with a system-scale multipronged approach must be tempered by failures that occur. The existence of non-responders and poor responders, and the obligate adherence to the protocol to sustain the arrest and reversal of cognitive decline suggest that there is a space for improvement in terms of the protocol’s effectiveness, potency, and specificity. Nevertheless, the apparent success of a systemic multimodal approach to treating AD in case studies strongly supports the systems network perspective on Alzheimer’s disease. On the other hand, the presented analysis validates the system-level programmatic approach and suggest novel avenues for its further development.

For example, given the apparently central and ambivalent roles of innate immunity and inflammation in AD pathobiology, stratifying patients on the basis of individual immunological profiles and addressing immune dysfunction and imbalances in an individualized, targeted manner may significantly enhance effectiveness of the current protocol. In this regard, differential immunomodulation by pharmaceuticals, nutraceuticals, and electroceuticals (e.g., vagus nerve stimulation), may be a worthy addition to the current therapeutic armamentarium.

Mapping out the entry portals and natures of peripheral stress, toxicity, and inflammation, and the routes by which they preferentially access the CNS in each individual patient may significantly improve differential diagnosis and, consequently, the precision and effectiveness of individualized treatment regimes.

Insufficient attention may have been paid to long-term maladaptive alterations that are acquired by affected cells, systems, and network domains during a decades-long prodromal phase, particularly by the neuroimmune system and its immune and neural components – the principal conduits interconnecting central and peripheral stress responses and inflammation. It is possible that the unconditional reversal and cure of AD may require the systematic identification and targeted reconstitution, rewiring, or reprogramming of misconfigured cells, systems, and network domains in each individual patient, with the purpose of restoring local and systemic homeostatic controls. Here, novel emerging therapeutic modalities such as immunomodulation [126], neuromodulation [127], and stem cell therapies [128] seem worthy of exploration.

The identification and treatment of major contributors to cognitive decline remains of highest priority. In this regard, certain common drivers of or contributors to cognitive decline may have been underappreciated either because they are hidden in plain sight or, on the contrary, well out of sight. Examples of the former are air pollution, chemical exposures, toxic molds, and other forms of chronic environmental toxicity, which is pervasive, insidious, unrelenting, highly diverse, and multi-natured [12, 124, 129]. Examples of the latter are the accumulated toxins and chronic clandestine infections such as latent, reactivating viral, bacterial, and fungal infections, intracellular infections, and polymicrobial biofilms [89, 130, 131].

## CONCLUSIONS

Reconciling multimodal clinical profiles of early-stage AD patients and research knowledge accumulated in diverse expert domains suggests that sporadic Alzheimer’s disease may not be a homogenous CNS disease, but a heterogeneous, system-level, network disorder, which is driven by chronic network stress and dyshomeostasis. It is hypothesized that the CNS and its key structures and circuits may become preferential targets of chronic systemic stress, toxicity, and inflammation mainly due to their central network positions and functions in the systems connectomics. Since chronic network stress and dyshomeostasis can be potentially driven by diverse endogenous and exogenous factors and their interactions, AD may have multiple etiologies and evolutionary trajectories that converge to a common clinicopathological endpoint recognized as Alzheimer’s disease.

The multiplicity of factors and mechanisms that can potentially drive CNS stress, neuroinflammation, and neurodegeneration, together with the multiplicity of evolutionary trajectories that converge to a common yet heterogeneous clinicopathological endpoint, may help to explain the daunting complexity and remarkable heterogeneity of AD [132–134], the inability of the field to move beyond statistical associations, continuing uncertainties over environmental and modifiable risk factors, and other persisting controversies [135].

The systems network AD perspective predicts that disease models and monotherapies that address a single network domain (a molecule, a cell type, an organ, or a system) or a single mechanism (e.g., β-amyloidosis, neuro inflammation, tauopathy, cholinergic signaling) are likely to be of limited utility in treating Alzheimer’s disease as a whole. Moreover, as different patients can have different disease drivers, etiologies, evolutionary trajectories, and endpoint configurations, their responses to a particular monotherapy are unlikely to be uniform. On the other hand, the systems network perspective suggests that addressing major contributors to chronic CNS distress and network dyshomeostasis with personalized, multimodal therapeutic programs on multiple network levels simultaneously and synergistically is likely to be significantly more effective than monotherapies in treating the syndrome as a whole. Indeed, as a proof of principle, a working prototype of such an approach has been created [116, 125] and validated in case studies, leading to sustained arrest and reversal of cognitive decline in early-stage AD patients [55].

The promising results of an integrative, systemic, precision medicine approach to treating Alzheimer’s disease suggests that evaluating and addressing the individual organism as a whole rather than focusing exclusively on an apparently failing part may represent a promising strategy to approach other complex chronic multifactorial disorders, which warrants further exploration and development.

## Appendix

This appendix provides brief functional descriptions of AD susceptibility factors, which have been conditionally assigned to three overlapping functional categories. The factors listed and their descriptions are intended to be representative rather than exhaustive.

### Innate immune reactivity and inflammatory response

Examples of AD susceptibility factors that belong to this group include NLRP1 (NACHT, LRR and PYD domains-containing protein 1), TLR4 (toll-like receptor 4), CD33 (Siglec-3) (a myeloid cell-surface receptor), TREM2 (triggering receptor expressed on myeloid cells 2), TREM1, MS4A6A and MS4A4AE (membrane-spanning proteins expressed on myeloid cells), CR1 (complement receptor 1), INPP5D (SHIP-1) (phosphatidylinositol (PtdIns) phosphatase), SORL1 (sortilin-related receptor L), ABCA7 (ATP-binding cassette transporter), PICALM (phosphatidylinositol-binding clathrin assembly protein), BIN1 (bridging integrator protein 1), and RIN3 (Ras and Rab interactor 3).

NLRP1 is a major sensor of intracellular pathogen- and damage/danger-associated molecular patterns (PAMPs and DAMPs, correspondingly). The sensor component of the NLRP1 inflammasome, NLRP1 is expressed widely across tissues, showing abundant expression in immune cells (neutrophils, monocytes/macrophages, dendritic cells, and B- and T-lymphocytes) and strong expression in epithelial cells lining the gastrointestinal and respiratory tracts, endometrial and endocervical glands, gallbladder, prostate, and breast [136]. NLRP1 genetic variants are associated with AD and a number of chronic peripheral inflammatory and autoimmune disorders [136, 137]. Emerging evidence implicates the inflammasome pathway in the initiation and/or progression of metabolic disorders and neurodegenerative diseases [138, 139]. Factors that trigger the inflammatory response via activation of inflammasomes can be divided into two classes, pathogen-associated and sterile. The first class includes microbial toxins, metabolites, and virulence factors. Factors that drive sterile inflammation include diverse crystalline and particulate materials and species, exemplified by exogenous materials such as silica, asbestos, and aluminum salts, and endogenous species such as cholesterol and urate crystals, Aβ, mutant SOD1, and prions [140–146].

TLR4 is a major pattern recognition receptor for diverse danger-associated extracellular ligands, which include lipopolysaccharide (LPS), several viral and bacterial components, and a variety of endogenous ligands such as oxidized low-density lipoproteins (oxLDLs), saturated fatty acids, Aβ, fibronectin, fetuin-A, β-defensins, and heat shock proteins [147]. TLR4 is predominantly expressed in cells of myeloid origin, which mainly comprise blood monocytes, tissue macrophages, and dendritic cells – the key initiators of tissue-specific immune responses. Engagement of TLR4 with cognate ligands triggers inflammatory response via activation of NF-κB signaling pathway and production of pro-inflammatory cytokines, chemokines, and other factors [147]. TLR4 is upregulated in conditions of tissue damage and inflammation, and may play a key role in the development of lipid-induced insulin resistance [148]. TLR4 polymorphisms have been associated with an increased risk of infections, and such conditions as tonsillitis, ulcerative colitis, Crohn’s disease, age-related macular degeneration, and Alzheimer’s disease [149–151].

CD33 is a canonical member of the sialic-acid-binding immunoglobulin-like lectins family, which are involved in cell-cell interactions and endocytosis. CD33-related Siglecs are predominantly expressed on innate immune cells and modulate cell reactivity upon recognition of cell surface sialic acids that act as “self-associated molecular patterns”. When engaged with cognate sialic-acid-containing ligands on other cells, most Siglecs suppress immune cell activation and production of inflammatory mediators by signaling via their ITIM-containing cytoplasmic domains and recruitment of inhibitory proteins such as INPP5D/SHIP-1 phosphatase. Furthermore, peripheral monocytes that carry the AD-associated CD33 mutation (rs3865444) have a decreased capacity for phagocytosis [73, 152].

TREM2 is a cell-surface receptor that initiates immune responses in tissue-specific macrophages, dendritic cells, and microglia. TREM2 has been also shown to regulate phagocytosis and suppression of inflammatory activity [72, 153]. Outside the CNS, TREM2 may play important roles in chronic obstructive pulmonary disease (COPD), gut injury, and infections [154].

CR1 is a complement receptor expressed on the surface of erythrocytes, monocytes, neutrophils, leukocytes, and glomerular podocytes, but not in the brain cells [13, 155]. CR1 functions as an immune adherence receptor, mediating the recognition and phagocytosis of cells, debris, and pathogens that have been opsonized by complement factors such as C1q, C3b, and C4b [155].

INPP5D (SHIP-1) acts as a negative regulator of B-cell antigen receptor signaling, myeloid cell proliferation/survival and chemotaxis, mast cell degranulation, and stress signaling in B-cells. INPP5D is also a key regulator of neutrophil migration [83].

RIN3 is a Ras effector and Rab5-directed guanine nucleotide exchange factor with functions in endocytosis, synaptic function, and immune responses. RIN3 is highly expressed and enriched in human mast cells. RIN3 may function as an inhibitor of mast cells migration to sites of infection and injury [71, 156].

SORL1 is a multifunctional endocytic, transport, and sorting receptor. SORL1 mediates the uptake of lipoproteins and proteases, and is involved in APP trafficking and sorting, SORL1 is highly expressed in the brain, particularly cerebellum, showing low to moderate expression in other tissues [74, 157].

ABCA7, PICALM, and BIN1 have functions in endocytosis [72, 73].

### Cell-cell and cell-matrix adhesions and communications

Examples of AD susceptibility factors that belong to this group include DSG2 (Desmoglein-2), ANK-1 (Ankyrin-1), NEDD9 (Neural Precursor cell expressed Developmentally Downregulated 9), CASS4 (Cas scaffolding protein family member 4), PTK2B (Protein-tyrosine kinase 2-beta / Focal adhesion kinase 2), CD2AP (CD2-associated protein), FERMT2 (fermitin family homolog 2), EPHA1 (Ephrin type-A receptor 1), APP (amyloid precursor protein), ADAM10 (a disintegrin and metalloproteinase 10), PSEN1(Presenilin-1), TREM1, TREM2, CD33, and HLA-DRB1/HLA-DRB5 (Major histocompatibility complex class II, DR beta 1/5).

The desmosomal cadherin DSG2 (desmoglein 2) is an integral component of intercellular junctions. DSG2 mediates functional cell-cell adhesions by linking plaque proteins and intermediate filaments. DSG2 is highly expressed in epithelial cells and cardiomyocytes. DSG2 is the primary high-affinity receptor used by adenovirus serotypes that cause respiratory tract infections. In epithelial cells, adenovirus binding to DSG2 triggers events reminiscent of epithelial-to-mesenchymal transition, leading to transient opening of intercellular junctions [158]. DSG2 also regulates β-catenin-mediated EMT signaling in pluripotent stem cells [159].

ANK-1 mediates the attachment of integral membrane proteins and adhesion molecules to the spectrin-actin membrane cytoskeleton, targeting them to specialized compartments and excitable membrane domains within the plasma membrane and endoplasmic reticulum. Ankyrins play a key role in integrating cells into tissues, and have functions in EMT [160, 161].

NEDD9 (a.k.a. CASS2) is a member of the Cas (Crk-associated substrate) scaffolding protein family. NEDD9 is a docking protein which plays a central coordinating role for tyrosine-kinase-based signaling related to cell adhesion. NEDD9 functions as a positive regulator of EMT, and is a metastasis marker in multiple cancers [85, 162].

CASS4 is another member of the Cas protein family of scaffolds. CASS4 is abundantly expressed in the lung and spleen, showing highest expression in leucocytes. CASS4 plays a role in cellular adhesion, cell migration, and motility by regulating integrity of focal adhesions and activity of focal adhesion kinases [163, 164].

PTK2B (a.k.a. FAK2, for focal adhesion kinase 2) is a non-receptor protein-tyrosine kinase that regulates reorganization of the actin cytoskeleton, cell polarization, cell migration, adhesion, and spreading. PTK2B is required for macrophage polarization and migration towards sites of inflammation. PTK2B has functions in EMT [165].

CD2AP is a scaffolding molecule that attaches cell surface receptors to the cytoskeleton. CD2AP is expressed ubiquitously, showing low levels in the brain and abundant expression in the kidneys and epithelial and endothelial cells [166]. CD2AP has functions in the formation of epithelial junctions and EMT ([167, 168]). CD2AP plays an important role in maintaining integrity and permeability of blood-brain barrier and other tissue barriers via control of adherence junctions [169, 170].

FERMT2 (a.k.a. kindlin-2 and Mig-2) is a member of the fermitin (kindlin) family of evolutionary conserved focal adhesion proteins. The best-known functions of fermitins are integrin activation and regulation of bidirectional integrin signaling and cell adhesion [171]. FERMT2 is expressed broadly and found in cardiomyocytes, endothelial cells, and fibroblasts. FERMT2 is highly expressed in endothelial cells, and is required for angiogenesis and blood vessel homeostasis. FERMT2 functions in cell-cell adherens junctions, and plays a role in wound healing and tissue repair. FERMT2 is overexpressed in cancers, where it promotes EMT and invasion. In tubular intestinal fibrosis of the kidney, a degenerative kidney disease, FERMT2 promotes progressive EMT in tubular epithelium [172].

EPHA1, a receptor tyrosine kinase, binds membrane-bound ephrin-A family ligands on adjacent cells to initiate contact-dependent bidirectional signaling between neighboring cells. Upon activation, EPHA1 induces cell attachment to the extracellular matrix, inhibiting cell spreading and motility. EPHA1 plays a role in angiogenesis and regulates cell proliferation. The EPH/ephrin-mediated bidirectional signaling has recently emerged as a major form of contact-dependent cell-cell communications that shape tissues during development and support the physiology and homeostasis of mature tissues [173, 174]. EPH receptors and ephrins are upregulated at sites of tissue injury and inflammation, and emerging evidence implicates EPH/ephrin signaling in the control of endothelial, blood-brain, and intestinal barrier permeability and EMT. Dysregulated EPH/ephrin signaling is implicated in a range of chronic inflammatory conditions [175–177].

APP is a cell surface receptor with key functions in axonal guidance, neuronal adhesions, and synaptogenesis. APP regulates neurite outgrowth through binding to components of the extracellular matrix such as heparin and collagen I and IV. In neuronal synapses, APP may regulate the balance between synapse-making (synaptoblastic) and synapse-breaking (synaptoclastic) signaling [178]. APP and classical cadherins, such as E- and N-cadherins, signal from the plasma membrane to the nucleus in a similar manner via release of C-terminal proteolytic fragments and share downstream effectors, such as evolutionary conserved YAP and TAZ transcription factors that control EMT [83, 179].

ADAM10 is a broad specificity membrane metalloprotease that cleaves and sheds extracellular domains of transmembrane proteins [180]. The list of ADAM10 substrates include diverse molecules involved in brain pathology, inflammation, and cancer [181]. Examples include Notch, APP, L-selectin, L1 adhesion molecule, N- and E-cadherins, TNF-alpha and IL6 receptors, Klotho, VEGF, and EGF. In neurons, ADAM10 provides the key alpha-secretase activity for proteolytic processing of APP. ADAM10 regulates epithelial cell-cell adhesion and migration by processing E-cadherin, and may have functions in EMT [182, 183]. ADAM10 also cleaves ephrins in the context of the EPH/ephrin complexes, thereby regulating cell-cell adhesions and communications [184].

Presenilin-1 (PSEN1) is a key component of the γ-secretase complex that catalyzes the intramembrane cleavage of integral membrane proteins. Presenilin-1 substrates are many, with most being type-I transmembrane proteins involved in the regulation of cell fate, adhesion, migration, neurite outgrowth, or synaptogenesis. Examples include Notch, APP, p75NTR, Desmoglein-2, N- and E-cadherins, SORL1, LRP1, IL1 and IL6 receptors, HLA, Klotho, VEGF, IGFR, insulin receptor, and ephrins and EPH receptors to name a few [185]; AD susceptibility factors are underlined). In cell adhesions, presenilin-1 associates with cadherins and plays a major role in the stabilization, dynamic turnover, maintenance, and dissolution of cell-cell adhesions.

The HLA-DRB1/HLA-DRB5 locus belongs to the major histocompatibility complex (MHC), a highly polymorphic region on chromosome 6 that encodes multiple proteins critical for immunity. The MHC polymorphisms are associated with infections, autoimmunity, cancer, and neuropathies, including AD, PD, and multiple sclerosis (MS) [73, 186]. Classically, MHC class II molecules are used by professional antigen-presenting cells (APCs), such as dendritic cells and macrophages, for presentation of exogenous antigens to CD4^+^ T helper cells [187]. Marked upregulation of MCH-II-expressing microglia has been demonstrated in many neurological disorders, including Alzheimer’s disease [188, 189]. MHCII^+^ microglia and CD4^+^ T cells accumulate during chronic neurodegeneration and reciprocally shape pathology [190].

More recent studies revealed however that APCs can also present processed and unprocessed antigens to other cell types. For example, perivascular dendritic cells capture and relay blood-derived antigens to mast cells via a release of exosomes carrying MCH-II-antigen complexes to elicit anaphylaxis [191]. In the gastrointestinal tract, dendritic cells capture and shuttle prions and potentially other antigens from the abluminal side of the intestines to highly innervated enteric lymphoid tissue, where prions are transferred from immune to neuronal cells via unknown mechanism(s), although exosomes and tunneling nanotubes are proposed as possible conduits [192, 193]. Moreover, MHC class II molecules are abundantly expressed on the surface of epithelial cells lining the gastrointestinal and respiratory tracts, presumably for non-professional presentation of transcytosed antigens to tissue-resident immune cells. MHC-II expression and transcytosis are upregulated in inflammatory conditions, upon exposure to inflammatory antigens, or in response to certain microbial products [194].

### Cell metabolism and detoxification

This group of AD susceptibility factors comprises proteins with functions in metabolic pathways that that are critical for proliferation, maintenance, and repair of cells and tissues, as well as for detoxification and antioxidant defenses. These include i) key enzymes of one-carbon metabolism, such as MTHFR (methylenetetrahydrofolate reductase), MTR (5-methyltetrahydrofolate-homocysteine methyltransferase, a.k.a. methionine synthase), MTRR (5-methyltetrahydrofolate-homocysteine methyltransferase reductase, a.k.a. methionine synthase reductase), and CBS (cystathionine β-synthase); ii) proteins involved in lipid transport and metabolism (ApoE, ApoJ, ABCA7, and SORL1); and iii) detoxifying enzymes such as COMT (catechol-*O*-methytransferase) and MAO-A (monoamine oxidase A), which catabolize reactive catechol and amine compounds, correspondingly.

Folate-dependent one-carbon metabolism (OCM) is a central hub of the basic cellular metabolism, which supports a large variety of metabolic pathways and reactions utilized in biosynthesis of proteins, lipids, nucleic acids, and neurotransmitters. OCM is essential for tissue and cell repair, cell proliferation, DNA repair, detoxification, and antioxidant defenses.

Uniquely studded with cofactors, such as heme, pyridoxal phosphate, and group B vitamins, one-carbon metabolism interlinks the folate cycle (folate assimilation), the methionine cycle (methionine conservation and generation of S-adenosylmethionine (SAM), the principal methyl donor), and the transsulfuration pathway (generation of glutathione, cysteine, and sulfate) via the intermediate homocysteine, a sulfur-containing amino acid [195].

Polymorphisms in OCM enzymes have been linked to a variety of chronic complex diseases, including AD and other neurological and psychiatric disorders. The associations of AD with decreased plasma folate, elevated plasma homocysteine, impaired SAM metabolism, and polymorphisms in OCM enzymes are well documented [196–198].

According to our observations, multiple inherited and acquired deficiencies in one-carbon metabolism (OCM), such as altered enzymatic activities and low levels of enzymatic co-factors (e.g., folate, B vitamins) are exceptionally common in AD patients, often in association with elevated plasma homocysteine levels. Targeted metabolic supplementation allows for correcting OCM deficiencies and reducing homocysteine levels, suggesting that these are therapeutically addressable factors [64, 125, 199]. Meta-analytical studies evaluating the role of OCM polymorphisms and B-vitamins status in AD are consistent with these clinical observations [200].

The best-known functions of ApoE, ApoJ, ABCA7, and SORL1 are in lipid metabolism and the trafficking and redistribution of lipids and cholesterol between and among different cells, cell types, and tissues. Chronic cell/tissue dyshomeostasis caused by chronic disease, toxicity, infections, and inflammation are associated with excessive production and accumulation of lipids, cholesterol, and lipophilic species in cells and tissues [201–203]. Recent studies suggest that export and redistribution of excessive lipids are mediated by HDL-like lipoprotein particles. Notably, in conditions of chronic inflammation, HDL-like particles acquire drastically altered composition and exhibit pro-inflammatory properties, in contrast to normal, anti-inflammatory HDL particles that mediates reverse cholesterol transport [204–207].

Apolipoprotein E epsilon 4 allele (ApoE4) is the single most significant genetic risk factor for sporadic Alzheimer’s disease. Having a single copy of ApoE4 gene more than doubles the risk of developing AD, whereas two copies of the gene increases the risk by 12-fold, as compared to the most common ApoE3 allele. Apolipoprotein E is a highly pleotropic protein involved multiple biological functions and processes, including neuronal growth and repair, nerve regeneration, lipid trafficking, and immune responses. ApoE has been implicated in longevity, atherosclerotic cardiovascular disease, evolution, inflammation, and development [70, 208–210]. The best-known function of ApoE is in lipid metabolism and transport, both in the CNS and on the periphery. ApoE is highly expressed in the liver, brain, and macrophages, where it mediates mobilization and redistribution of cholesterol and lipids [70]. ApoE and ApoJ (a.k.a. clusterin (CLU)), are the principal apolipoproteins that manage trafficking and redistribution of cholesterol and lipids in the brain [211, 212]. ApoE and ApoJ are also major stress factors secreted at the sites of tissue injury to scavenge spilled cholesterol, lipids, lipophilic species, and denatured proteins from extracellular environments. The extracellular ApoE can constitute up to 5% of total soluble protein at the sites of injured and regenerating peripheral nerves [70]. ApoE and ApoJ, a generic cell stress factor, are among the primary chaperons for removing β-amyloid from the brain [135, 213, 214]. Multiple studies linked ApoE4 with enhanced innate immune reactivity and poor outcomes in CNS and systemic infections [215–220]. A recent study showed that ApoE4 might act as a transcription factor that regulates expression of multiple genes associated with synaptic function, programmed cell death, microtubule disassembly, trophic support, aging, and insulin resistance [210].

COMT (catechol-*O*-methyltransferase) methylates endogenous and xenobiotic catechol compounds, targeting them for degradation and clearance. COMT substrates include epinephrine, norepinephrine, catechol-estrogens (e.g., E2), and drugs (e.g., levodopa). COMT regulates estrogen levels in tissues, and plays a central role in regulating dopamine levels in the prefrontal cortex.

MAO-A (monoamine oxidase A) catalyzes oxidative deamination of monoamines, such as serotonin, melatonin, dopamine, epinephrine, and norepinephrine, generating reactive aldehydes, ammonia, and hydrogen peroxide. Both excess and insufficiency of MAO-A activity are associated with a wide range of neurological and psychiatric disorders, including AD, aggression, antisocial behavior, panic disorder, bipolar disorder, and major depressive disorder. MAO-A inhibitors are used for treating depression and PD.

MAO-A and COMT are best known as enzymes that catabolize neurotransmitters and catechol-estrogens. However, these are generic detoxification enzymes that are abundantly expressed in many tissues outside the CNS, where they act on diverse amine and catechol compounds of endogenous and exogenous origins. The role of monoamine oxidases and COMT in metabolism of endogenous and xenobiotic compounds outside the CNS may have been underappreciated [221].

## Footnotes

* Early-stage AD patients are operationally defined to include individuals with subjective cognitive impairment (SCI), mild cognitive impairment (MCI), and patients with moderate AD who retained most of the activities of daily living, in accord with the clinical criteria outlined in Recommendations from the National Institute on Aging-Alzheimer's Association workgroups on diagnostic guidelines for Alzheimer's disease (Alzheimers Dement. 2011 May; 7(3): 263-292). Whenever possible, evaluation of functional and structural AD biomarkers by brain imaging (amyloid-PET, FDG-PET, and/or MRI), CSF amyloid and tau (ATI), and cognitive assessment (MoCA, and/or MMSE) were performed to corroborate AD diagnosis.
